# Comparison of different scoring systems for predicting in-hospital mortality for patients with Fournier gangrene

**DOI:** 10.1007/s00345-023-04552-3

**Published:** 2023-08-14

**Authors:** Yufi Aulia Azmi, Firas F. Alkaff, Johan Renaldo, Soetojo Wirjopranoto, Rinta Prasetiyanti, Kevin Muliawan Soetanto, Sovia Salamah, Abdul Khairul Rizki Purba, Maarten J. Postma

**Affiliations:** 1 Department of Urology, Faculty of Medicine, Universitas Airlangga-Dr. Soetomo General Academic Hospital, Surabaya, Indonesia; 2 Department of Health Sciences, University of Groningen, University Medical Center Groningen, Groningen, The Netherlands; 3 Division of Pharmacology and Therapy, Department of Anatomy, Histology, and Pharmacology, Faculty of Medicine Universitas Airlangga, Surabaya, Indonesia; 4 Division of Nephrology, Department of Internal Medicine, University Medical Center Groningen, Groningen, The Netherlands; 5 Department of Clinical Pathology, Faculty of Medicine, Universitas Airlangga-Dr. Soetomo General Academic Hospital, Surabaya, Indonesia; 6 Department of Immunology, Faculty of Medicine Siriraj Hospital, Mahidol University, Bangkok, Thailand; 7 Department of Public Health and Preventive Medicine, Faculty of Medicine Universitas Airlangga, Surabaya, Indonesia; 8 Institute of Science in Healthy Ageing and Healthcare (SHARE), University Medical Center Groningen, University of Groningen, Groningen, The Netherlands; 9 Unit of Pharmacotherapy, Epidemiology and Economics (PTE2), Department of Pharmacy, University of Groningen, Groningen, The Netherlands; 10 Department of Economics, Econometrics and Finance, Faculty of Economics and Business, University of Groningen, Groningen, The Netherlands

**Keywords:** Diagnosis, Fournier gangrene, Hospital mortality, Indonesia, Infectious disease

## Abstract

**Purpose:**

To compare different scoring systems for predicting in-hospital mortality in patients with Fournier gangrene (FG).

**Methods:**

A comprehensive literature search was performed to find all scoring systems that have been proposed previously as a predictor for in-hospital mortality in patients with FG. Data of all patients with FG who were hospitalized in one of Indonesia’s largest tertiary referral hospitals between 2012 and 2022 were used. The receiver operating characteristic (ROC) curve analysis was performed to evaluate the diagnostic performance of the scoring systems.

**Results:**

Ten scoring systems were found, i.e., Fournier’s Gangrene Severity Index (FGSI), Uludag FGSI, simplified FGSI, NUMUNE Fournier score (NFS), Laboratory Risk Indicator for Necrotizing Fasciitis, age-adjusted Charlson comorbidity index, sequential organ failure assessment (SOFA), quick SOFA, acute physiology and chronic health evaluation II, and surgery APGAR score (SAS). Of 164 FG patients included in the analyses, 26.4% died during hospitalization. All scoring systems except SAS could predict in-hospital mortality of patients with FG. Three scoring systems had areas under the ROC curve (AUROC) higher than 0.8, i.e., FGSI (AUROC 0.905, 95% confidence interval (CI) 0.860–0.950), SOFA (AUROC 0.830, 95% CI 0.815–0.921), and NFS (AUROC 0.823, 95% CI 0.739–0.906). Both FGSI and SOFA had sensitivity and NPV of 1.0, whereas NFS had a sensitivity of 0.74 and an NPV of 0.91.

**Conclusion:**

This study shows that FGSI and SOFA are the most reliable scoring systems to predict in-hospital mortality in FG, as indicated by the high AUROC and perfect sensitivity and NPV.

**Supplementary Information:**

The online version contains supplementary material available at 10.1007/s00345-023-04552-3.

## Introduction

Fournier gangrene (FG) is a polymicrobial infection caused by necrotizing fasciitis involving the perineal and genital areas [1]. This infection can occur not only in men but also in women and children [2, 3]. The prevalence of FG varied among countries, with a higher prevalence in developing countries than in developed countries because of the poor hygiene, lower socioeconomics, and lower education levels [4]. Due to its rapid spread, the mortality rate of FG is high. Globally, the mortality rates range from 20% to 40% [4, 5].

Mortality in FG can be avoided with adequate resuscitation, swift surgical debridement, and admission to critical care [6]. Therefore, there is a need for a scoring system that can be used as a prognostic index for patients with FG, so that aggressive treatments can be started early. Several scoring systems have been proposed to predict in-hospital mortality in patients with FG [7, 8]; however, no study comparing the diagnostic performance of all proposed scoring systems has been conducted to this date. Thus, this study aimed to evaluate the diagnostic performance of all scoring systems that have been proposed to predict mortality in patients with FG. To this end, we used data from Indonesia, a country with high rate of in-hospital mortality among patients with FG [4, 9].

## Materials and methods

### Ethics approval

The study was conducted according to the principles provided in the Declaration of Helsinki and was approved by the ethical review board of Dr. Soetomo General Academic Hospital (Approval no. 0911/LOE/301.4.2/V/2022 on May 25, 2022). The requirement of written informed consent was waived because of the retrospective nature of this study, with only data from medical records being used.

### Search strategy for the available scoring systems

A comprehensive literature search was performed to identify all scoring systems that have been proposed for predicting in-hospital mortality in patients with FG. The search was conducted in three different databases, namely Science Direct, PubMed, and Scopus, on April 12, 2023. The following search terms were used: (“Fournier gangrene”) AND (“Scoring System” OR “Score” OR “Questionnaire” OR “Prognostic”).

### Study design and population

This retrospective observational study was conducted at Dr. Soetomo General Academic Hospital in Surabaya, Indonesia. This hospital is one of the largest tertiary referral hospitals in Indonesia and is a referral center for the eastern part of Indonesia. The study population was all patients diagnosed with FG, who were hospitalized between January 2012 and December 2022. Patients with incomplete data were excluded from the analyses.

### Data collection

To complete all the scoring systems, comorbidities, sociodemographic and laboratory evaluation data, and outcomes were collected from the medical records. FG was diagnosed based on the presence of pain, erythema, ulcers, swelling, crepitus, necrosis, and purulent discharge found in the emergency room and confirmed by tissue inspection in the operating room. The evaluated outcome was in-hospital mortality, defined as death during the hospital stay.

### Statistical analyses

Statistical analyses were conducted using IBM SPSS version 26.0 (IBM Corp., Armonk, NY, USA) and R version 4.2.1 (R Foundation for Statistical Computing, Vienna, Austria). Data normality was determined using the one-sample Kolmogorov–Smirnov test. Data were presented as mean ± standard deviation for normally distributed data, as median [interquartile range (IQR)] for skewed data, and as frequency (valid percentage) for nominal data. Receiver operating characteristic (ROC) curve analysis was performed to test the diagnostic performance of the scoring systems. Differences between the groups were tested using the independent *t* test, Mann–Whitney *U* test, and χ^2^ test, depending on the data type and data distribution. A two-tailed *p* value < 0.05 was considered statistically significant for all analyses.

## Results

### Literature search results

From the comprehensive search, 10 scoring systems have been proposed to predict in-hospital mortality in patients with FG. Four of the scoring systems were developed specifically for FG (FG severity index (FGSI)) [10], Uludag FGSI (UFGSI) [11], simplified FGSI (SFGSI) [12], and NUMUNE Fournier score (NFS) [13]), whereas the other six (Laboratory Risk Indicator for Necrotizing Fasciitis (LRINEC) [14], age-adjusted Charlson comorbidity index (aCCI) [8], sequential organ failure assessment (SOFA) [15] , quick SOFA (qSOFA) [4], acute physiology and chronic health evaluation (APACHE) II [11], and surgical APGAR score (SAS) [8] ) were not FG-specific. The parameters used for the scoring system and the proposed cutoff score are presented in Supplementary Table 1.

### Baseline characteristics of the study population

In total, 164 patients with FG were admitted to the hospital between January 2012 and December 2022, and all of them were included in the analyses. The median patient age was 52 [42–61] years, and the majority of them were men. The median length of hospital stay was 11 [5–21] days. During hospitalization, 43 (26.2%) patients died. There were no significant differences in age and sex between survivors and non-survivors. Non-survivor group had a shorter hospital stay and were more often reported to have diabetes as comorbidity. Furthermore, the C-reactive protein and serum creatinine levels were significantly higher in the non-survivor group than in the survivor group (Table 1).

**Table 1:** Baseline characteristics of the study population

Variables	Total (*N* = 164)	Survivors (*N* = 121)	Non-survivors (*N* = 43)	*p* Value
Age (years)	52 [42–61]	52 [43–60]	55 [41–62]	0.3
Sex, *n* (%)				0.8
Male	151 (92.1)	111 (73.5)	40 (26.5)	
Female	13 (7.9)	10 (76.9)	3 (23.1)	
Location, *n* (%)				0.3
Perineum	50 (30.5)	35 (70)	15 (30)	
Scrotum	91 (55.5)	66 (72.5)	25 (27.5)	
Penoscrotal	23 (14)	20 (87)	3 (13)	
Length of stay (days)	11 [5–21]	12 [7–23]	7 [3–17]	0.003
Diabetes mellitus, *n* (%)	99 (60.4)	67 (55.4)	32 (74.4)	0.028
Hypertension, *n* (%)	40 (24.4)	31 (25.6)	9 (26.2)	0.5
Systolic blood pressure (mmHg)	110 [70–150]	115 [80–150]	87 [70–122]	< 0.001
Mean arterial pressure (mmHg)	82 [69–92]	88 [78–94]	68 [64–69]	< 0.001
Heart rate (x/min)	88 [50–120]	84 [55–119]	108 [50–120]	< 0.001
Respiratory rate (x/min)	20 [16–29]	18 [16–24]	27 [18–29]	< 0.001
C-reactive protein (mg/dL)	10.6 [4.23–20.85]	8.1 [3.6–17.83]	14.5 [10.6–26.2]	0.004
Glucose (mmol/L)	127 [96–201]	122 [93–201]	132 [103–217]	0.4
Hemoglobin (g/dL)	11.25 ± 2.09	11.4 ± 2.07	10.8 ± 2.14	0.1
White blood cells (10^3^/µL)	16.28 [11.86–21.65]	16.02 [12.05–19.5]	17.69 [11.1–26.6]	0.2
Sodium level (mmol/L)	135 [131–139]	135 [132–139]	134 [ 130–138]	0.5
Serum creatinine (mg/dL)	1.1 [0.8–1.54]	0.99 [0.78–1.3]	1.5 [1–2.1]	< 0.001
Potassium level (mmol/L)	4 [3.6–4.5]	3.9 [3.6–4.5]	4.2 [3.6–4.6]	0.4
Hematocrit (%)	34.21 ± 6.77	34.62 ± 6.86	33.08 ± 6.44	0.2

### Comparison of different scoring systems

**Fig. 1:**
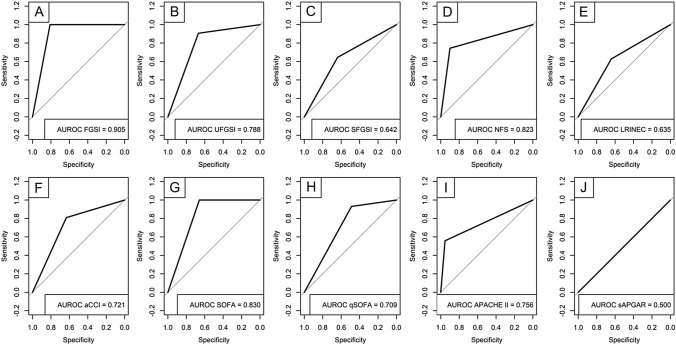
Area under the receiver operating characteristic curves (AUROC) of different scoring systems for predicting in-hospital mortality in patients with FG. **A** Fournier gangrene severity index (FGSI), **B** Uludag FGSI, **C** simplified FGSI, **D** NUMUNE Fournier score, **E** laboratory risk indicator for necrotizing fasciitis, **F** age-adjusted Charlson comorbidity index, **G** sequential organ failure assessment (SOFA), **H** quick SOFA, **I** acute physiology and chronic health evaluation II, and **J** surgery APGAR score

The median score of all scoring systems is presented in Table 2. Compared to the survivor group, the non-survivor group had higher scores in all scoring systems (all *p* value < 0.001). The ROC analysis showed that all scoring systems with the proposed cutoff could be used to predict in-hospital mortality for patients with FG (all *p* value < 0.05), except for SAS (*p* = 1.0). Among all scoring systems, three scoring systems had AUROC > 0.800, i.e., FGSI (AUROC 0.905, 95% CI 0.860–0.950), SOFA (AUROC 0.830, 95% CI 0.815–0.921), and NFS (AUROC 0.823, 95% CI 0.739–0.906) (Table 3). The ROC analysis of all scoring systems is visualized in Fig. 1.

**Table 2:** The score from different scoring systems

Scoring systems	Total (*N* = 164)	Survivors (*N* = 121)	Non-survivors (*N* = 43)	*p* Value
FGSI, median [IQR]	8 [5–12]	6 [4–8]	14 [12–15]	< 0.001
UFGSI, median [IQR]	8 [6–12]	7 [5–9]	13 [11–14]	< 0.001
SFGSI, median [IQR]	1 [0–3]	1 [0–2]	3 [0–4]	< 0.001
NFS, median [IQR]	1 [1–3]	1 [1–1]	2 [1–3]	< 0.001
LRINEC, median [IQR]	5 [3–7]	4 [3–6]	6 [4–9]	< 0.001
aCCI, median [IQR]	1 [1–2]	1 [0–1]	2 [1–2]	< 0.001
SOFA median [IQR]	2 [0–18]	1 [0–9]	9 [4–18]	< 0.001
qSOFA, median [IQR]	2 [1–2]	2 [1–2]	3 [2–3]	< 0.001
APACHE II, median [IQR]	3 [0–24]	2 [0–13]	15 [19–24]	< 0.001
SAS, median [IQR]	8 [7–8]	7 [7–8]	8 [8–9]	< 0.001

*aCCI* Age-adjusted Charlson comorbidity index; *APACHE II* acute physiology and chronic health evaluation II; *FGSI* Fournier gangrene severity index; *LRINEC* laboratory risk indicator for necrotizing fasciitis; *NFS* NUMUNE Fournier score; *qSOFA* quick SOFA; *SAS* surgery APGAR score; *SFGSI* simplified FGSI; *SOFA* sequential organ failure assessment; *UFGSI* Uludag FGSI

**Table 3:** Receiver operating characteristic curve analyses and diagnostic performance of the evaluated scoring systems to predict in-hospital mortality in patients with Fournier gangrene

Scoring system	Cutoff score	AUROC	95% CI	p value	Sensitivity	Specificity	PPV	NPV
FGSI	> 9	0.905	0.860–0.950	< 0.001	1.0	0.81	0.65	1.0
UFGSI	≥ 9	0.788	0.714–0.862	< 0.001	0.91	0.67	0.49	0.95
SFGSI	> 2	0.642	0.540–0.743	0.006	0.47	0.82	0.48	0.81
NFS	> II	0.823	0.739–0.906	< 0.001	0.74	0.90	0.73	0.91
LRINEC	≥ 6	0.635	0.543–0.737	0.006	0.63	0.65	0.39	0.83
aCCI	≥ 4	0.721	0.644–0.816	< 0.001	0.79	0.67	0.46	0.90
SOFA	≥ 4	0.830	0.815–0.921	< 0.001	1.0	0.74	0.57	1.0
qSOFA	≥ 2	0.709	0.628–0.790	< 0.001	0.93	0.49	0.39	0.95
APACHE II	≥ 13	0.756	0.666–0.860	< 0.001	0.56	0.97	0.86	0.86
SAS	≤ 4	0.500	0.399–0.601	1.000	1.0	0.0	0.26	–

*95% CI* 95% confidence interval; *aCCI* age-adjusted Charlson comorbidity index; *APACHE II* acute physiology and chronic health evaluation II; *AUROC* area under the receiver operating characteristic curve; *FGSI* Fournier gangrene severity index; *LRINEC* laboratory risk indicator for necrotizing fasciitis; *NFS* NUMUNE Fournier score; *NPV* negative predictive value; *PPV* positive predictive value; *qSOFA* quick SOFA; *SAS* surgery APGAR score; *SE* standard error; *SFGSI* simplified FGSI; *SOFA* sequential organ failure assessment; *UFGSI* Uludag FGSI

The diagnostic performance of each scoring system, i.e., sensitivity, specificity, positive predictive value (PPV), and negative predictive value (NPV), is presented in Table 3. FGSI and SOFA had perfect sensitivity and NPV, whereas APACHE II had the highest specificity and PPV among other scoring systems.

## Discussion

This study compared the diagnostic performance of 10 scoring systems that have been proposed previously as a predictor for in-hospital mortality, i.e., FGSI, UFGSI, SFGSI, NFS, LRINEC, aCCI, SOFA, qSOFA, APACHE II, and SAS, using data from Indonesia. In this study, the scoring system with the highest AUC was FGSI, followed by SOFA and APACHE II. In regards to the diagnostic performance, FGSI and SOFA had the highest sensitivity and NPV, whereas APACHE II had the highest specificity and PPV.

Theoretically, a scoring system is expected to have high sensitivity, specificity, PPV, and PPV, and an AUC nearing 1.0. However, one scoring system meeting all these demands is rare. Therefore, depending on the expected performance, a scoring system does not necessarily need to be highly sensitive and specific at the same time. If the intended use is to screen for an event with low prevalence, high specificity and PPV are more important. By contrast, sensitivity and NPV must be emphasized if finding an event with high prevalence is its main objective [17, 18]. Since in-hospital mortality is prevalent in patients with FG [4, 5], screening modalities should have high sensitivity and NPV. Accordingly, our results showed that either FGSI or SOFA is the most suitable scoring system for predicting in-hospital mortality of patients with FG.

Three previous studies have also evaluated the diagnostic performance of FG scoring systems in Indonesia. Putra et al. (2020) evaluated FGSI and SFGSI in 34 patients with FG between 2013 and 2017, using the same cutoff as our study [19]. Similar to our finding, this study found that FGSI has a higher AUROC value than SFGSI. In addition, our study and the previous study had comparable AUROC values. However, our findings were in contrast with those of two other studies by the same research group [4, 20]. In the first study, Noegroho et al. (2021) included 69 patients from one center in Indonesia between 2013 and 2017 and found that FGSI has lower AUROC value than qSOFA, albeit the sensitivity and NPV were quite comparable with our findings [4]. In the second study, Noegroho et al. (2021) included 83 patients from one center in Indonesia between 2015 and 2019 and found that FGSI had a AUROC value of 0.842 [20]. The differences between the present study and these previous studies might be explained by the cutoff that was used for the FGSI. The present study used the cutoff of ≤ 9 vs. > 9, whereas Noegroho et al. (2021) used the cutoff of < 9 vs. ≥ 9 in both studies [4, 19]. When FGSI was first developed, the proposed threshold value was 9 (≤ 9 vs. > 9) [10].

Several studies from different countries have tried to validate the diagnostic performance of FGSI. A study from India reported that by using the same cutoff, FGSI had a AUROC of 0.96 and a sensitivity of 0.917 [21]. Other studies have also showed similar findings [22, 23]. By contrast, studies from Turkey reported that the AUROC of FGSI with the same cutoff was below 0.9 and that the diagnostic performance of UFGSI was better [11, 24]. This suggested that the geographical area may influence the diagnostic performance of the FGSI.

While the diagnostic performance of qSOFA was inferior compared to FGSI, SOFA showed the contrary. The AUROC, sensitivity, and NPV of SOFA were comparable to those of FGSI. Our finding was similar to the study that initially proposed the use of SOFA as the prognostic scoring system for patients with FG [15]. SOFA has been previously reported to be a good predictor of in-hospital mortality in other life-threatening conditions such as infection, heart failure, and COVID-19 [16, 25, 26]. In addition, SOFA was also reported to be a good predictor of primary wound closure in patients with FG [27].

Roghmann et al. (2012) previously recommended the use of aCCI and SAS for daily practice instead of FGSI, as these two scoring systems had good AUROC, more easily calculated at the bedside, generally applicable, and well validated [8]. When the same cutoff (≥ 4 for aCCI and ≤ 4 for SAS) was applied, we found a similar AUROC value in regard to the aCCI, but not for the SAS. Nevertheless, the AUROC value of aCCI in our study was far lower (> 0.1 in difference) than that of FGSI or SOFA.

This study has two important limitations. Although this study was conducted in a tertiary referral hospital, this was a single-center study. In addition, since this was a retrospective study using data from medical records, several factors such as differences in treatment between patients could not be controlled. Despite these limitations, this study was the first to compare all the proposed scoring systems for predicting in-hospital mortality in patients with FG. Moreover, this study has the greatest number of patients thus far. The largest single-center study in the literature only included 120 patients [4, 28]

In summary, we found that FGSI and SOFA are the most reliable scoring systems to predict in-hospital mortality in patients with FG, as indicated by the high AUROC and perfect sensitivity and NPV. Therefore, we recommend that all patients with FG who are hospitalized should be immediately assessed with FGSI or SOFA, so that whether aggressive treatment should be given or not, can be decided as early as possible. Future multi-institutional studies across different countries and continents are needed to confirm our study findings.

### Supplementary Information

Below is the link to the electronic supplementary material.

Supplementary file1 (PDF 272 KB)

## Data Availability

The data that support the findings of this study are available from the corresponding author (YAA) upon reasonable request.
