# Supplementary material for: Comparison of different scoring systems for predicting in-hospital mortality for patients with Fournier gangrene
Source: World J Urol. 2023 Aug 14;41(10):2751–7. doi: 10.1007/s00345-023-04552-3 (PMC10581919; doi:10.1007/s00345-023-04552-3)
Supplement: Supplementary file 1 — Supplementary file1 (PDF 272 KB) [file 345_2023_4552_MOESM1_ESM.pdf]

Supplementary Tables

Supplementary Table 1. Measured parameters and cutoff score of each scoring systems from the literature

Scoring system	Number of parameters	Parameters	Cutoff score
FGSI	9	Temperature, heart rate, respiratory rate, serum sodium, serum potassium, serum creatinine, hematocrit, leucocyte counts, and serum bicarbonate	>9 [9]
UFGSI	11	Age and dissemination score in addition to the measured parameters from FGSI	≥9 [11]
SFGSI	3	Serum creatinine, hematocrit, and serum potassium	>2 [12]
NFS	5	Age, serum urea, red cell distribution width, serum albumin, and presence of sepsis	>II [13]
LRINEC	6	C-reactive protein, white blood cell counts, hemoglobin, serum sodium, serum creatinine, and blood glucose	≥6 [10]
aCCI	20	Age and presence of the following diseases: myocardial infarction, congestive heart failure, peripheral vascular disease, cerebrovascular disease, dementia, chronic pulmonary disease, rheumatic disease, peptic ulcer, liver disease, diabetes mellitus, hemiplegia, kidney disease, malignancy, leukemia, lymphoma, and acquired immunodeficiency syndrome	≥4 [15]

SOFA	6	Oxygenation, platelet counts, Glasgow coma scale, serum bilirubin, blood pressure or the use of vasoactive drugs, and serum creatinine or urine output per day	≥4 [16]
qSOFA	3	Blood pressure, respiratory rate, and Glasgow coma scale	≥2 [4]
APACHE II	11	Temperature, blood pressure, heart rate, respiratory rate, oxygenation, arterial pH, serum potassium, serum natrium, serum creatinine, hematocrit, white blood cell counts, and Glasgow coma scale	≥13 [10]
SAS	3	Estimated blood loss, heart rate, and blood pressure	≤4 [14]

aCCI, age-adjusted Charlson Comorbidity Index; APACHE II, Acute Physiology and Chronic Health Evaluation II; FGSI, Fournier gangrene severity index; LRINEC, Laboratory Risk Indicator for Necrotizing Fasciitis; NFS, NUMUNE Fournier score; qSOFA, quick SOFA; SAS, Surgery APGAR score; SFGSI, simplified FGSI; SOFA, Sequential Organ Failure Assessment; UFGSI, Uludag FGSI
